# Real-time feature extraction of P300 component using adaptive nonlinear principal component analysis

**DOI:** 10.1186/1475-925X-10-83

**Published:** 2011-09-23

**Authors:** Arjon Turnip, Keum-Shik Hong, Myung-Yung Jeong

**Affiliations:** 1 Department of Cogno-Mechatronics Engineering, Pusan National University; 30 Jangjeon-dong, Geumjeong-gu, Busan 609-735, Korea; 2 School of Mechanical Engineering, Pusan National University; 30 Jangjeon-dong, Geumjeong-gu, Busan 609-735, Korea

## Abstract

**Background:**

The electroencephalography (EEG) signals are known to involve the firings of neurons in the brain. The P300 wave is a high potential caused by an event-related stimulus. The detection of P300s included in the measured EEG signals is widely investigated. The difficulties in detecting them are that they are mixed with other signals generated over a large brain area and their amplitudes are very small due to the distance and resistivity differences in their transmittance.

**Methods:**

A novel real-time feature extraction method for detecting P300 waves by combining an adaptive nonlinear principal component analysis (ANPCA) and a multilayer neural network is proposed. The measured EEG signals are first filtered using a sixth-order band-pass filter with cut-off frequencies of 1 Hz and 12 Hz. The proposed ANPCA scheme consists of four steps: pre-separation, whitening, separation, and estimation. In the experiment, four different inter-stimulus intervals (ISIs) are utilized: 325 ms, 350 ms, 375 ms, and 400 ms.

**Results:**

The developed multi-stage principal component analysis method applied at the pre-separation step has reduced the external noises and artifacts significantly. The introduced adaptive law in the whitening step has made the subsequent algorithm in the separation step to converge fast. The separation performance index has varied from -20 dB to -33 dB due to randomness of source signals. The robustness of the ANPCA against background noises has been evaluated by comparing the separation performance indices of the ANPCA with four algorithms (NPCA, NSS-JD, JADE, and SOBI), in which the ANPCA algorithm demonstrated the shortest iteration time with performance index about 0.03. Upon this, it is asserted that the ANPCA algorithm successfully separates mixed source signals.

**Conclusions:**

The independent components produced from the observed data using the proposed method illustrated that the extracted signals were clearly the P300 components elicited by task-related stimuli. The experiment using 350 ms ISI showed the best performance. Since the proposed method does not use down-sampling and averaging, it can be used as a viable tool for real-time clinical applications.

## Background

The first recording of the electric field of a human brain was made by the German psychiatrist Hans Berger in Jena, Germany, in 1924. He named the recorded signals electroencephalograms (EEGs) [1]. Over the past few decades, this signal has attracted very considerable interest and attention in the study of cognitive processes in both clinical [2-9] and research areas [10-16]. Its main advantages are non-invasive measurement, superior temporal resolution, easy implementation, and low cost [17,18]. An event-related potential (ERP), as a derivative of the EEG, is a measured brain response directly resulted from a thought or perception. In 1964 and 1965, respectively, two groups (Chapman and Bragdon [19] and Sutton et al. [20]) independently discovered a P300 component (a wave peak approximately 300 milliseconds (ms) after a task-relevant stimulus). Recently, a great variety of potential applications of the ERP-based P300 component have been widely studied [21-26].

Ideally, the EEG machine records, along the scalp, the electrical activities generated by the firing of neurons within the brain. The present problem is that EEG signals contain the neurons' activities located in some significant distances away from the sensors (electrodes). Therefore, given the distance between the electrode and the neuronal activities, the EEG signal collected at any point on a person's scalp is a nonlinear mixture of the activities generated over a large brain area. In this paper, the recorded EEG data are assumed to be a linear mixture of neuronal activities for brevity. Certainly, dealing with the typical low-amplitude and low signal-to-noise ratio (SNR) potentials, the removal of other biological signals becomes one of the major challenges in the study of ERPs. To resolve this problem, down-sampling and averaging methods of EEG data over multiple trials are usually required. However, the down-sampling method can cause some signals to become indistinguishable and distorted, which implies an alteration of the original characteristics of the waveform of information. Also, the averaging method assumes that the signals are long-time stationary and deterministic relative to the stimulus onset. This assumption might cause the loss of time resolution specifically for dissimilar trials. Also, the stationarity and determinacy assumption on EEG signals might not work, because one must consider other factors such as maturation, age, sex, state-of-consciousness, psychiatric and neurological disorders, etc [27].

In this paper, a more efficient means of feature extraction is developed to cope with the drawbacks of the down-sampling and averaging method. Previous research has shown that several aspects of the ERP (especially the latency, magnitude, and topography) are highly variable across trials [27,28]. Many techniques [29-33] appeared in research area to resolve the problem of EEG (specifically for obtaining P300 components) are not sufficiently standardized for clinical usage. Moreover, those techniques usually have been performed off-line. In this paper, a real-time feature extraction method for P300 components using an adaptive nonlinear principal component analysis (ANPCA) incorporating the multilayer neural network (MNN) is proposed. The MNN technique has been widely adopted in the fields of information and neural sciences (i.e., feature extraction, classification, modeling, etc.) [34-39]. The experimental results in this paper show that the implementation of the proposed method achieves a very significant statistical improvement in extracting P300 components.

The main contributions of this paper are the following. (i) The developed multi-stage principal component analysis (PCA) applied at the pre-separation step reduces external noises and artifacts significantly, and separates the colored source in the measured EEG signals. (ii) The designed adaptive rule in the whitening step makes the subsequent separation algorithm to converge fast. (iii) The combination of the proposed ANPCA method and the MNN for feature extraction can identify the P300 components in real-time (i.e., without down-sampling and averaging). (iv) Furthermore, the proposed method can become a viable tool in both research and clinical applications.

## Methods

### Data acquisition

**Figure 1:**
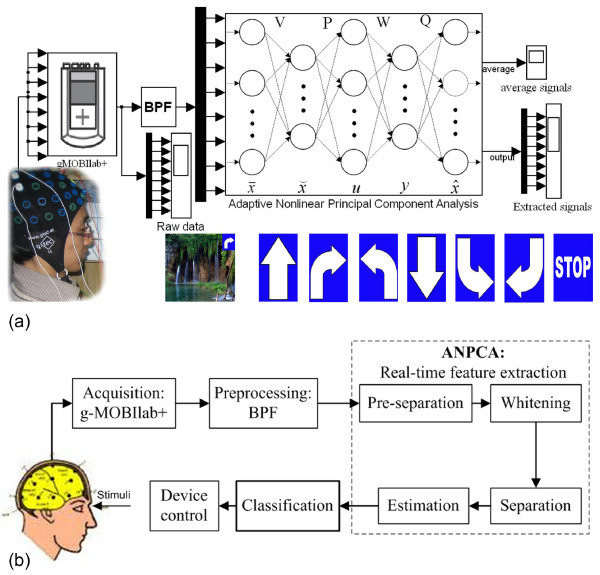
**The proposed scheme for real-time feature extraction (the seven traffic signals flash one at a time to evoke P300): (a) the overall scheme, (b) block diagram**. The configurations of the ANPCA algorithm incorporated with the MNN scheme for real-time feature extraction of the independent components according to the P300 component.

Figures 1(a) and 1(b) show the overall schematic and block diagram, respectively, of the proposed real-time feature extraction method. In the experiment, two masters students and five Ph.D. students (all males, age 32 ± 5 years, none of whom had any known neurological deficits) have participated. A seven-choice signal paradigm (i.e., forward, turn right, turn left, backward, backward right, backward left, and stop) is used to stimulate the seven subjects. They sit in a comfortable chair in front of a computer monitor located at 60 cm away from their eyes. The subjects are asked to count silently the number of times of the flashes of a preselected image on the screen while imagining a car moving in the direction of the flashed signal. Four seconds after a starting tone, seven different images flash in random order, one image at a time. A software program (E-Prime 2.0, developer: Schneider, Sharpsburg-USA) is employed for presenting stimuli.

**Figure 2:**
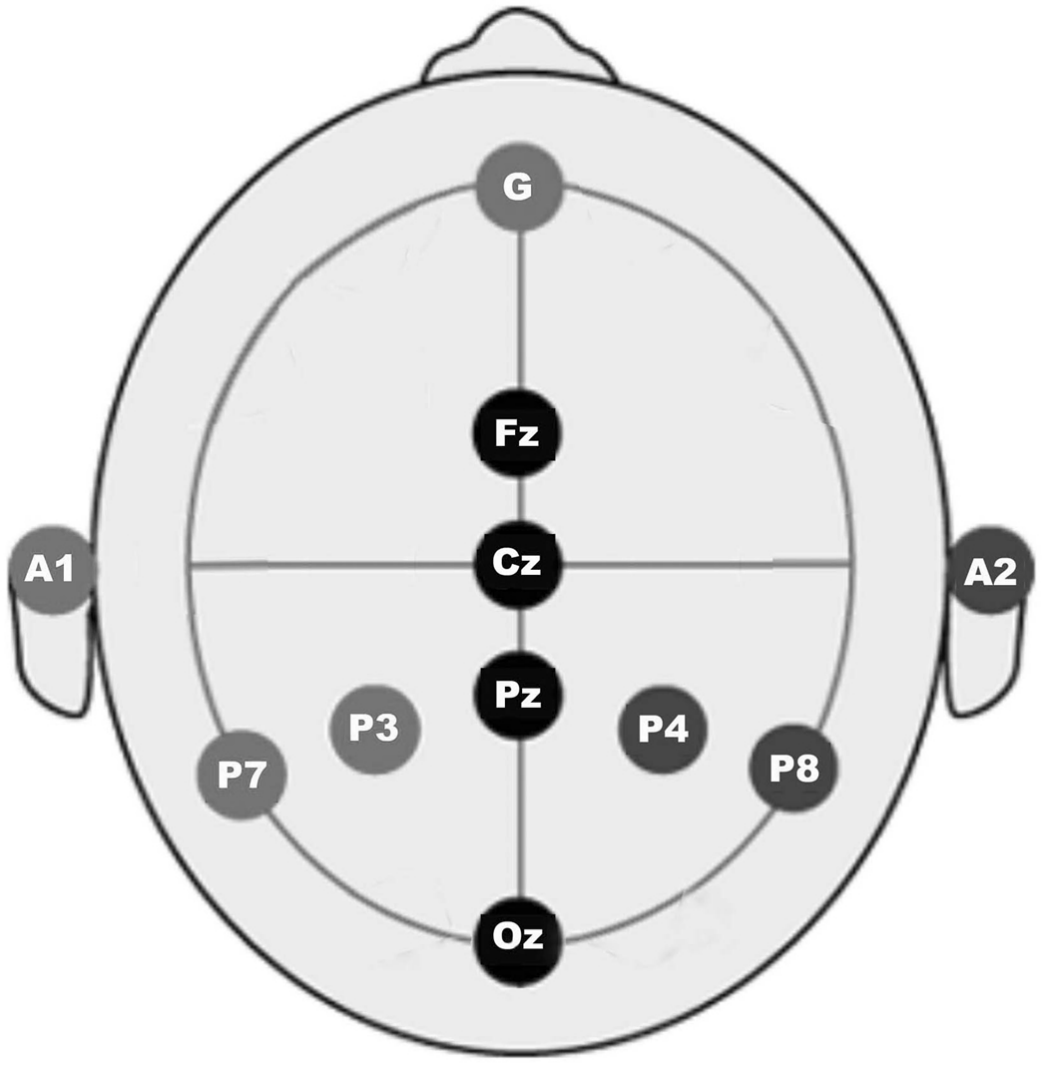
**The eight-electrodes configuration**. The standard positions (i.e., Fz, Cz, Pz, Oz, P7, P3, P4, and P8) prescribed by the 10-20 International System with a linked-ears reference.

The left-hand side box in Figure 1(a) shows a g-MOBIlab+ biosignal acquisition device (Christoph Guger, Austria), with which the EEG signals are recorded continuously and digitized at a 256 Hz sampling rate. Figure 2 depicts the positioning of the eight electrodes (channels) at Fz, Cz, Pz, Oz, P7, P3, P4, P8 by following the 10-20 International System [40] and the linked-ears reference. The ground electrode is placed at the center of the forehead. The impedance at each location is kept below 5 kΩ. The participants are supposed not to have any eye and head movements during the EEG recording. Each subject records four sessions; four different image-flash durations (i.e., 25 ms, 50 ms, 75 ms, and 100 ms, respectively) followed by a 300 ms blank screen. Hence, the inter-stimulus intervals (ISIs) in this work range from 325 ms to 400 ms.

### Real-time feature extraction

Let *M *be the number of measured EEG signals and *N *be the number of unknown input sources. Then, the measured signal at channel *i*, *x*_*i*_(*k*), can be represented as a linear combination of *N *unknown mutually statistically independent source signals *s_j_*(*k*), *j *= 1,2, ..., *N*, as follows (typically *M *≥ *N*) [41,42].

(1) $$x_{i}\left(k\right)=\;\overset{N}{\underset{j=1}{\sum }}a_{ij}s_{j}\left(k\right)+n_{i}\left(k\right),$$

or in matrix form,

(2) x(k)=As(k)+n(k),

where *x*(*k*) = [*x*_1_(*k*), *x*_2_(*k*), ..., *x_M_*(*k*)]*^T^*∈ R^*M *^is the vector of EEG signals, *A *∈ R^*M *× *N *^with entries *a_ij _*is the unknown *M *× *N *mixing matrix, *s*(k) = [*s*_1_(*k*), *s*_2_(*k*), ..., *s_N_*(*k*)]*^T^*∈ R^N ^is the unknown vector of colored source signals, and *n*(*k*) ∈ R^*M *^is the vector of additive noises. The objective of this work is to estimate both *A *and *s*(*k*). The following assumptions are made: Individual components of the source vector *s*(*k*) are statistically independent of one another; the matrix *A *is invertible and has full rank; each component in *s*(*k*) is a stationary; and the noise vector *n*(*k*) is white with Gaussian distribution. The P300 extraction is made in the following steps: pre-separation, whitening, separation, and estimation without ignoring the additive noise signal *n*(*k*).

#### Pre-separation step

The pre-separation step uses a multi-stage PCA to separate the sources and also to reduce external noises and artefacts from the measured signal vector. The eigenvalue decomposition of the correlation matrix *R_xx _*of the measured signal *x*(*k*) is given by [42]

(3) $$R_{xx}=E\left\{x\left(k\right)x^{T}\left(k\right)\right\}=V\Lambda^{}V^{T},$$

where *Λ *∈ R^*M × N *^is a pseudo-diagonal matrix. On the basis of the largest eigenvalues, the spatial whitening procedures can be written as

(4) $$\bar{x}\left(k\right)=\;Bx\left(k\right)=\Lambda_{j}^{-1∕2}V_{j}^{T}x\left(k\right),$$

where *Λ_j _*= diag{*λ_1_*, *λ_2_*, ..., *λ_N_*} with *λ_1 _*≥ *λ_2 _*≥ ... ≥ *λ_N _*and *V_j _*= {*v*_1_, *v*_2_,...*v_N_*} ∈ R^*N×M*^. Therefore, the PCA is performed for a new vector of signals, which is defined [41,42]

(5) $$\tilde{x}\left(k\right)=\;\bar{x}\left(k\right)+\bar{x}\left(k-\tau \right),$$

where *τ *is an arbitrary time delay. The covariance matrix of the vector $\tilde{x}\left(k\right)$ is expressed as

(6) $$\begin{array}{c} R_{\tilde{x}\tilde{x}}=R_{\tilde{x}}\text{(0)}=E\text{\{}\tilde{x}\text{(}k\text{)}{\tilde{x}}^{T}\text{(}k\text{)\}}\\ =\text{2}R_{\bar{x}}\left(0\right)+R_{\bar{x}}\left(\tau \right)+R_{\bar{x}}^{T}\left(\tau \right), \end{array}$$

where $R_{\bar{x}\bar{x}}=R_{\bar{x}}\text{(0)}=E\text{\{}\bar{x}\text{(}k\text{)}{\bar{x}}^{T}\text{(}k\text{)\}}=HR_{ss}H^{T}=I$, under the assumption that *H *= *BA *is orthogonal and *R_SS _*= *I *and

(7) $$R_{\bar{x}}\left(\tau \right)=E\left\{\bar{x}\left(k\right){\bar{x}}^{T}\left(k-\tau \right)\right\}=HR_{s}\left(\tau \right)H^{T}.$$

Hence, the matrix decomposition can be written

(8) $$R_{\tilde{x}\tilde{x}}=HD\left(\tau \right)H^{T}=V_{\tilde{x}}\Lambda_{\tilde{x}}V_{\tilde{\text{x}}}^{\text{T}},$$

where *D*(*τ*)is a diagonal matrix expressed as

(9) $$D\text{(}\tau \text{)}=\text{2}I+R_{s}\left(\tau \right)+R_{s}^{T}\left(\tau \right),$$

with diagonal elements *d_ii_*(*τ*) = 2(1+*E*{*s_i_*(*k*)*s_i_*(*k*-*τ*)}) If the diagonal elements are distinct, the eigenvalue decomposition is unique. Thus, the mixing matrix and the input vector $\overset{⌣}{x}\left(k\right)$, respectively, can be estimated as $A=B^{+}V_{\tilde{\text{x}}}$ and

(10) $$\overset{⌣}{x}\left(k\right)=V_{\tilde{x}}^{T}\bar{x}\left(k\right)=V_{\tilde{x}}^{T}Bx\left(k\right).$$

Assume that the process $\overset{⌣}{x}\left(k\right)\in {\text{C}}^{M}$ comprises a zero-mean sequence whose covariance matrix is defined as in (3), and that we are going to extract its complex-values eigenvectors *v_i _*and corresponding principal components (PCs) in real-time. Employing a self-supervising principle and hierarchical neural network architecture, the PCs(${\overset{⌣}{x}}_{i}$) are extracted sequentially as

(11) $${\overset{⌣}{x}}_{i}=v_{i}^{T}x=\overset{M}{\underset{p=1}{\sum }}v_{ip}x_{p}\left(t\right).$$

The vector *v_i _*should be determined in such a way that the reconstructed vector $\bar{x}=v_{i}^{*}{\overset{⌣}{x}}_{i}$ will reproduce the input vector $\overset{⌣}{x}\left(t\right)$ according to a suitable optimization. For this purpose, let us define a complex-valued instantaneous error vector as

(12) $$\begin{array}{c} e_{i}\left(t\right)={\left[e_{i1}\left(t\right),e_{i2}\left(t\right),\ldots ,e_{iM}\left(t\right)\right]}^{T}\\ =x\left(t\right)-\bar{x}\left(t\right)=x\left(t\right)-v_{i}^{*}\overset{⌣}{x}\left(t\right)\\ =\left(\text{I}-v_{i}v_{i}^{H}\right)x\left(t\right)=e_{i}^{R}\left(t\right)+je_{i}^{I}\left(t\right), \end{array}$$

where *I *is the identity matrix, $e_{i}^{R}\left(t\right)$ and $e_{i}^{I}\left(t\right)$ are the real part and imaginary parts of the error vector *e_i_*(*t*), respectively, and $j=\sqrt{-1}$. In order to find the optimal value of the vector *v_i_*, we can define the following standard 2-norm cost function.

(13) $$\begin{array}{c} E_{i}\left(v_{i}\right)=\frac{1}{2}\left({∥e_{i}^{R}∥}_{2}^{2}+{∥e_{i}^{I}∥}_{2}^{2}\right)\\ =\frac{1}{2}\left(\overset{M}{\underset{p=1}{\sum }}{\left(e_{ip}^{R}\right)}^{2}+\overset{M}{\underset{p=1}{\sum }}{\left(e_{ip}^{I}\right)}^{2}\right), \end{array}$$

where $e_{ip}^{R}$ is the *p*th element of $e_{i}^{R}$. The minimization of the cost function (13), according to the standard gradient descent approach for the real and imaginary parts of the vector $v_{i}=v_{i}^{R}+jv_{i}^{I}$, leads to a set of differential equations as

(14) $$\begin{array}{c} \frac{dv_{ip}^{R}}{dt}=-\beta_{i}\frac{\partial E_{i}\left(v_{i}\right)}{\partial v_{ip}^{R}}\\ =\beta_{i}\left(E_{1}+x_{p}^{R}\overset{M}{\underset{h=1}{\sum }}E_{2}+x_{p}^{I}\overset{M}{\underset{h=1}{\sum }}E_{3}\right), \end{array}$$

(15) $$\begin{array}{c} \frac{dv_{ip}^{I}}{dt}=-\beta_{i}\frac{\partial E_{i}\left(v_{i}\right)}{\partial v_{ip}^{I}}\\ =\beta_{i}\left(E_{4}+x_{p}^{R}\overset{M}{\underset{h=1}{\sum }}E_{3}-x_{p}^{I}\overset{M}{\underset{h=1}{\sum }}E_{2}\right), \end{array}$$

where *β_i _*> 0 is the learning rate, $E_{1}=e_{ip}^{R}{\overset{⌣}{x}}_{i}^{R}+e_{ip}^{I}{\overset{⌣}{x}}_{i}^{I}$, $E_{2}=e_{ih}^{R}v_{ih}^{R}-e_{ih}^{I}v_{ih}^{I}$, $E_{3}=e_{ih}^{R}v_{ih}^{I}+e_{ih}^{I}v_{ih}^{R}$, $E_{4}=e_{ip}^{R}{\overset{⌣}{x}}_{i}^{I}-e_{ip}^{I}{\overset{⌣}{x}}_{i}^{R}$, ${\overset{⌣}{x}}_{i}\overset{\Delta }{=}{\overset{⌣}{x}}_{i}^{R}+j{\overset{⌣}{x}}_{i}^{I}$, and $e_{ip}\overset{\Delta }{=}e_{ip}^{R}+je_{ip}^{I}$. Combining (14) and (15) and taking into account that $v_{ip}\overset{\Delta }{=}v_{ip}^{R}+jv_{ip}^{I}$, the adaptation law for updating the parameters is obtained as

(16) $$\begin{array}{c} \frac{dv_{ip}\left(t\right)}{dt}=\beta_{i}\left(t\right)\left({\overset{⌣}{x}}_{i}\left(t\right)e_{ip}^{*}\left(t\right)+\right)\\ \left(x_{p}^{*}\left(t\right)\sum_{h=1}^{M}v_{ih}\left(t\right)e_{ih}\left(t\right)\right), \end{array}$$

which can be written in matrix form as

(17) $$\frac{dv_{i}}{dt}=\beta_{i}\left[{\overset{⌣}{x}}_{i}e_{i}^{*}+x^{*}v_{i}^{T}e_{i}\right],$$

for any *v_i_*(0) ≠ 0, *β_i_*(*t*) > 0. Since the second term in (17), which can be written $x^{*}v_{i}^{T}e_{i}=x^{*}\left(1-v_{i}^{H}v_{i}\right){\overset{⌣}{x}}_{i}$, tends quickly to zero as $v_{i}^{H}v_{i}$ tends to 1 with *t*→∝ it can be neglected. The adaptation law in (17) can be further simplified to

(18) $$\begin{array}{c} \frac{dv_{i}}{dt}=\beta_{i}{\overset{⌣}{x}}_{i}e_{i}^{*}=\beta_{i}{\overset{⌣}{x}}_{i}{\left[x-v_{i}^{*}{\overset{⌣}{x}}_{i}\right]}^{*}\\ =\beta_{i}v_{i}^{T}x\left[I-v_{i}v_{i}^{H}\right]x^{*}, \end{array}$$

where (.)* denotes a complex conjugate. In discrete time, the adaptation law in (18) can be written

(19) $$v_{i}\left(k+1\right)=v_{i}\left(k\right)+\beta_{i}\left(k\right){\overset{⌣}{x}}_{i}\left(k\right)E_{5},$$

where $E_{5}=\left[x^{*}\left(k\right)-v_{i}\left(k\right){\overset{⌣}{x}}_{i}^{*}\left(k\right)\right]$.

#### Whitening step

The whitening step uses the PCA to transform the data into an appropriate space and to reduce the redundancy of the observed data. The separated input vector $\tilde{x}\left(k\right)$ is whitened in the second step by applying the following transformation.

(20) $$u\left(k\right)=\;P\text{(}k\text{)}\overset{⌣}{x}\left(k\right),$$

where *u*(*k*) is the whitened *k *vector, and *P *is the whitening matrix, which is determined using the neural learning approach. The objective is to find a simple adaptive algorithm for estimating the whitening matrix *P*, such that the covariance matrix of the whitened signals *u*(*k*) will be a diagonal matrix, that is, *R_uu _*= *E*{*uu^T^*} = diag{*λ*_1_, *λ*_2_, ..., *λ*_N_} = *I_N_*, and will be mutually uncorrelated if all of the cross-correlations are zero, that is, *r_ij _*= *E*{*u_i_u_j_*} = 0, for all *i *≠ *j*, with non-zero autocorrelations $r_{ij}=E\left\{u_{i}^{2}\right\}=\lambda_{i}>0$. Therefore, the minimization function can be formulated in the following 2-norm.

(21) $$\begin{array}{c} J_{2}\left(W\right)=\frac{1}{4}\overset{N}{\underset{i=1}{\sum }}\overset{M}{\underset{j=1}{\sum }}{\left(E\left\{u_{i}u_{j}\right\}-\lambda_{i}\delta_{ij}\right)}^{2}\\ =\frac{1}{4}{∥E\left\{uu^{T}\right\}-I_{N}∥}^{2}. \end{array}$$

To derive an adaptive learning algorithm, the following transformation

(22) $$\begin{array}{c} E\left\{uu^{T}\right\}=E\left\{P\overset{⌣}{x}{\overset{⌣}{x}}^{T}P^{T}\right\}\\ =E\left\{PAss^{T}{\text{(}PA\text{)}}^{T}\right\}\\ =BR_{ss}B^{T}=BB^{T}, \end{array}$$

is used, where *B *= *PA *is the global transformation matrix from *s *to *u*. Without loss of generality, *R_ss _*= *E*{*ss^T^*} = *I_N _*is assumed. By substituting (22) into (21), the optimization criterion can be written as

(23) $$\begin{array}{c} J_{2}\left(W\right)=\frac{1}{4}{∥BB^{T}-I_{N}∥}^{2}\\ =\frac{1}{4}tr\left[\left(BB^{T}-I_{N}\right)\left(BB^{T}-I_{N}\right)\right]. \end{array}$$

Applying the standard gradient descent approach and the chain rule, the derivative of (23) is obtained as

(24) $$\frac{dB}{dt}=\eta \left(I_{N}-BB^{T}\right)B=\eta \left(I_{N}-R_{uu}\right)B.$$

Taking into account that *B *= *PA *and assuming that *A *varies very slowly in time (i.e., *dA*/*dt*≈0), we have

(25) $$\frac{dP}{dt}=\eta \left(I_{N}-R_{uu}\right)P.$$

Using the simple Euler formula, the corresponding discrete-time adaptive learning algorithm can be written as

(26) $$P\text{(}k+\text{1)}=P\text{(}k\text{)}+\eta \left(k\right)\left(I_{N}-R_{uu}^{k}\right)P\text{(}k\text{)},$$

where *η*(*k*) is the learning parameter to be adjusted according to $\eta \left(k\right)=1∕\left\{\xi ∕\left(\eta \left(k-1\right)\right)+{∥u\left(k\right)∥}_{2}^{2}\right\}$, and *ξ *is the forgetting factor (i.e., 0 <*ξ *< 1). The covariance matrix *R_uu _*can be estimated as

(27) $${\hat{R}}_{uu}^{k}=\langle uu^{T}\langle =\frac{1}{N}\overset{N-1}{\underset{k=0}{\sum }}{u\left(k\right)u\left(k\right)}^{T},$$

where $u\left(k\right)=P\left(k\right)\overset{⌣}{x}\left(k\right)$.

#### Separation step

The separation of the whitened signals *u*(*k*) is the third step of the proposed algorithm, which is accomplished by applying the nonlinear principal component analysis (NPCA) learning rule. The multichannel linear separation transformation is given in the following form.

(28) $$y\left(k\right)=\;W^{T}\text{(}k\text{)}u\left(k\right),$$

where *W*(*k*) is the separation matrix, whose values are updated through the NPCA learning rule. If the independent signals are zero-mean, the generalized covariance matrix of *f*(*y_i_*) and *g*(*y_j_*) (*f*(*y_i_*) and *g*(*y_j_*) are different and odd nonlinear activation functions such that *f*(*y*) = *y*^3 ^and *g*(*y*) = tanh(*y*)) is a non-singular diagonal matrix *R_fg _*= *E*{*f*(*y*)*g^T^*(*y*)}*-E*{*f*(*y*)}*E*{*g^T^*(*y*)}. On the basis of the independence criterion, the nonlinear covariance matrix is given as [41,43]

(29) $$R_{fg}=\langle f\left(y\right)g^{T}\left(y\right)\langle +I,$$

where *f*(*y*) = [*f*(*y*_1_), *f*(*y*_2_), ..., *f*(*y_N_*)]*^T ^*and *g*(*y*) = [*g*(*y*_1_), *g*(*y*_2_), ..., *g*(*y_N_*)]*^T^*, provided that *E*{*f*(*y_i_*)} = 0 or *E*{*g*(*y_i_*)} = 0. To satisfy these conditions for arbitrary distributed sources, the nonlinearities are selected as *f_i_*(*y_i_*) = *φ_i_*(*y_i_*), *g_i_*(*y_i_*) = *y_i _*or *f_i_*(*y_i_*) = *y_i_*, *g_i_*(*y_i_*) = *φ_i_*(*y_i_*), where *φ_i_*(*y_i_*) are suitably designed nonlinear functions, defining *g*(*y*) as an odd function and *f*(*y*) = *g*(*y*)-*y*. Therefore, similarly to (21)-(26), a real-time implementation algorithm can be derived as

(30) $$W\left(k+1\right)=\;W\left(k\right)-\mu \left(k\right)f\left(y\left(k\right)\right)g^{T}yW\left(k\right),$$

where *g^T^y *= (*f^T^y*(*k*)-*y^T^*(*k*)). Since the separation matrix *W*(*k*) is assumed to be orthogonal (i.e., *W^T^*(*k*)*W*(*k*) = *I*), the real-time adaptation rule can be rewritten as

(31) $$W\left(k+1\right)=\;W\left(k\right)+\mu \left(k\right)f\left(y\left(k\right)\right)W_{b}\;W\left(k\right),$$

where *y*(*k*) is the separated signal and the output of the second step, *W_b _*= (*u^T^*(*k*)-*f^T^y*(*k*)), * μ*(*k*) is the learning parameter (it is adjusted according to $\mu \left(k\right)=1∕\left\{\gamma ∕\left(\mu \left(k-1\right)\right)+{∥y\left(k\right)∥}_{2}^{2}\right\}$ with the forgetting factor 0 <*γ *< 1), and *f*(.)is a suitably chosen nonlinear function that is usually selected to be odd in order to ensure both stability and signal separations. These nonlinear functions require the use of high-order statistics (HOS). In the present study, *f*(.)was chosen as *f*(*t*) = tanh(*t*). Finally, since *f*(*t*) = *dg*(*t*)/*dt*, *g*(*t*) = In[cosh(*t*)].

#### Estimation step

The final step is the estimation of the independent component basis vector of the mixing matrix *A*(*k*). The estimate of the observed data is given by

(32) $$\hat{x}\left(k\right)=Q\text{(}k\text{)}y\left(k\right).$$

Comparing (32) with (2), and since ŝ(k)≅y(k) (i.e., ŝ(k) is the estimated source signal *s*(*k*)), it can be concluded that Â(k)=Q(k). Therefore, the columns of the matrix *Q*(*k*) are the estimates of the columns of the matrix Â(k). Since *Q*(*k*) is the estimation matrix, its values (similarly to (26)) are updated through the adaptation law as

(33) $$Q\left(k+1\right)=\;Q\left(k\right)+\alpha \left(k\right)Q_{e}y^{T}\left(k\right),$$

where $Q_{e}=\left[\hat{\text{x}}\left(k\right)-Q\left(k\right)y\left(k\right)\right]$. The quality of the source estimate in *y*(*k*) can be measured using the zero-forcing solution. Such a solution attempts to adapt the demixing matrix such that

(34) $$\underset{k\to \infty }{\lim}C\left(k\right)Â\text{(}k\text{)}=\Phi D,$$

where *C*(*k*) = *W*(*k*)*P*(*k*)*V*(*k*), *Φ *is a (*M *× *M*) permutation matrix with one unity entry in any row or column, and *D *is a diagonal nonsingular scaling matrix. In this case, it becomes

(35) $$y_{i}\left(k\right)=d_{jj}s_{j}\left(k\right)+\overset{N}{\underset{l=1}{\sum }}b_{il}\left(k\right)n_{l}\left(k\right),$$

for some non-replicative assignment *j*→*i *for 1 ≤ *i *≤ *N *and 1 ≤ *j *≤ *M *Thus, each element of *y*(*k*) is the sum of a single unique source in *s*(*k*) and a noise term. In each simulation run, the performance index (*PI*) is evaluated using the following equation [44].

(36) $$PI\left(k\right)=\frac{1}{M-1}\left(M-\frac{1}{2}\overset{N}{\underset{i=1}{\sum }}\left(C_{a}+C_{b}\right)\right),$$

where $C_{a}=\frac{\underset{1\leq j\leq M}{\max}{\left|c_{ij}\left(k\right)\right|}^{2}}{\sum_{j=1}^{M}{\left|c_{ij}\left(k\right)\right|}^{2}}$, $C_{b}=\frac{\underset{1\leq j\leq M}{\max}{\left|c_{ji}\left(k\right)\right|}^{2}}{\sum_{j=1}^{M}{\left|c_{ji}\left(k\right)\right|}^{2}}$, *c_ij _*denotes the (*i, j*)th element of the matrix in *C*(*k*), corresponding to the *j*th independent component (IC) in the desired subset of sources. This dimensionless performance metric measures the deviation of the combined system from a diagonally scaled permutation matrix (i.e., 0 ≤ *PI*(*k*) ≤ 1 for all matrices *C*(*k*), *PI*(*k*) is one when the sources maximally mixed in the outputs, and *PI*(*k*) is zero when the desired subset of the ICs is perfectly separated). The first term in (36) gives the error of the separation of the output component *y_i_*(*k*) in (35) with respect to the sources and the second term measures the degree of the desired IC, *c_j_*, appearing multiple times at the output. The integration of the four steps is called the adaptive nonlinear principal component analysis (ANPCA) method. In order to improve the flexibility, efficiency, and performance of blind signals separation or extraction, the proposed ANPCA scheme is run upon a multilayer neural network. The multiple layers of neurons with nonlinear transfer functions allow the network to learn both linear and nonlinear relationships between input and output vectors. Furthermore, this allows us to combine second-order statistics (SOS) and the HOS algorithm to extract features having different statistical properties, existing at various layers, and originating from various sources. The synaptic weights in each layer are updated by employing the algorithm described above.

## Results

**Figure 3:**
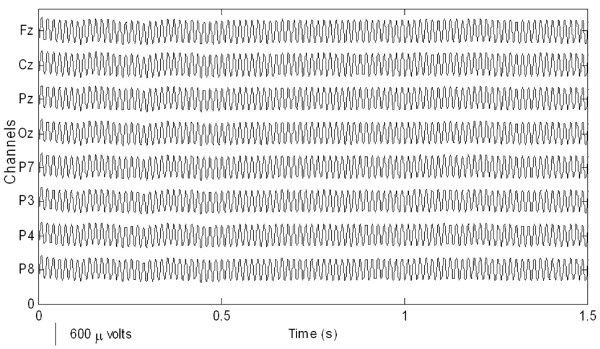
**The measured raw EEG signals**. The EEG signals recorded continuously and digitized at a 256 Hz sampling rate using a g-MOBIlab^**+ **^biosignal acquisition device.

**Figure 4:**
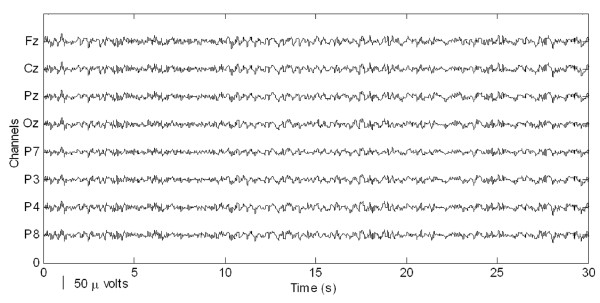
**The pre-processed EEG signals that were band-passed**. The EEG signals were pre-processed using a sixth-order BPF with cut-off frequencies of 1 Hz (i.e., to remove the trend from low frequency bands) and 12 Hz (i.e., to remove unimportant information from high frequency bands).

**Figure 5:**
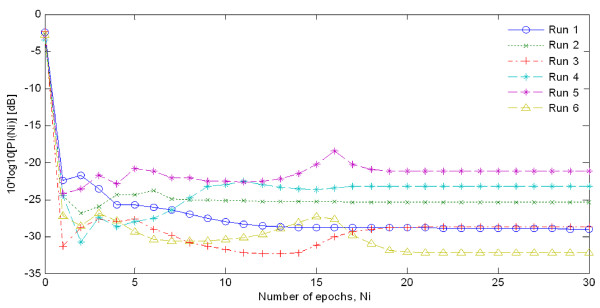
**Evolutions of *PI*(*Ni*) for six different runs of the ANPCA algorithm using 350 ms ISl**. The performance of the ANPCA algorithm in (31) was evaluated using (35), where ***W***(0) = ***I***. A single block of ***N ***= 7000 samples has been used to compute all coefficient updates for six run, where $\hat{x}\left(k+Ni\right)=\hat{x}\left(k\right)$ for all integer values i ≥ 0 and *i *≤ *k *≤ *N*-1.

**Figure 6:**
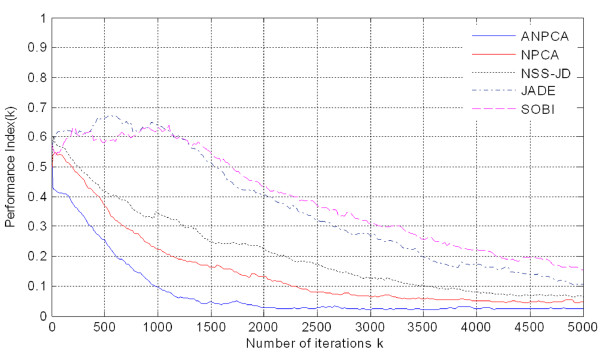
**Comparison of separation performance indices: the proposed method (ANPCA) vs. other algorithms (NPCA, NSS-JD, JADE, and SOBI)**. To evaluate the robustness of the ANPCA against background noise, the separation performance indices of the ANPCA is compared with others well known algorithms (i.e., NPCA, NSS-JD, JADE, and SOBI algorithms).

**Figure 7:**
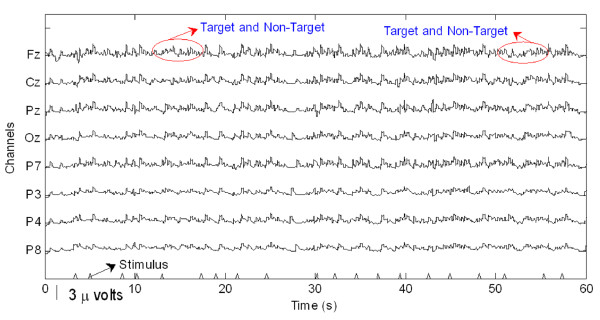
**Extracted P300 components in real time (325 ms ISI)**. For the ISI of about 325 ms, it was found that the amplitude of the P300 component was higher than for the other ISI but noisier than for the higher ISI.

**Figure 8:**
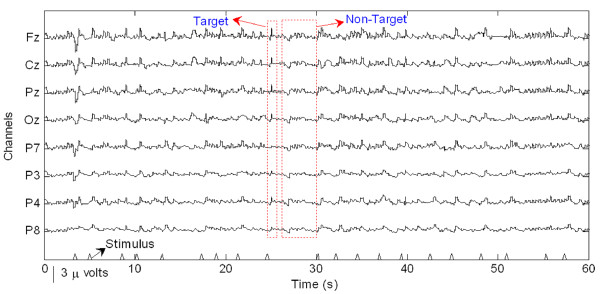
**Extracted P300 components in real time (350 ms ISI)**. For the ISI of about 350 ms, the target and non-target amplitudes were clearer and easier to distinguish than for the other ISI.

**Figure 9:**
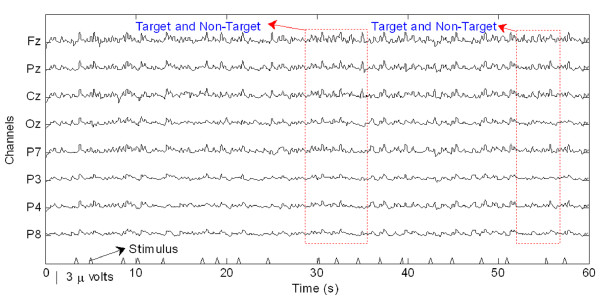
**Extracted P300 components in real time (375 ms ISI)**. For the ISI of about 375 ms, it was found that in some sessions the non-target amplitudes were higher than the target ones.

**Figure 10:**
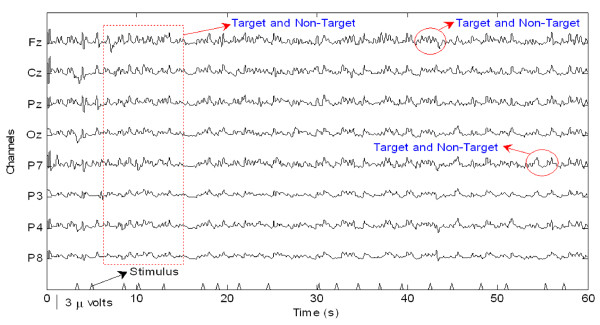
**Extracted P300 components in real time (400 ms ISI)**. For the ISI of about 400 ms, it was found that none of the channels showed similar behaviour.

**Figure 11:**
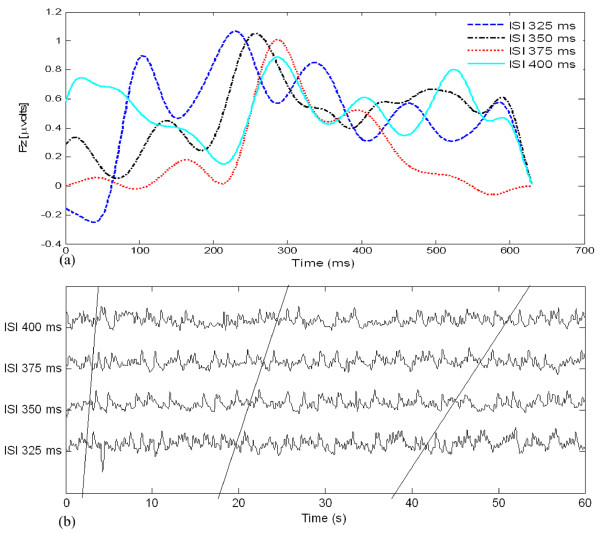
**Comparison of P300 amplitudes for four different ISIs (Fz channel): (a) P300 amplitude upon a single stimulus, (b) P300 upon multiple stimuli**. The amplitudes of the P300 component for each ISI, which indicates that the short ISI could increase both the target and non-target amplitudes. The ISI of about 350 ms shows better performance.

**Figure 12:**
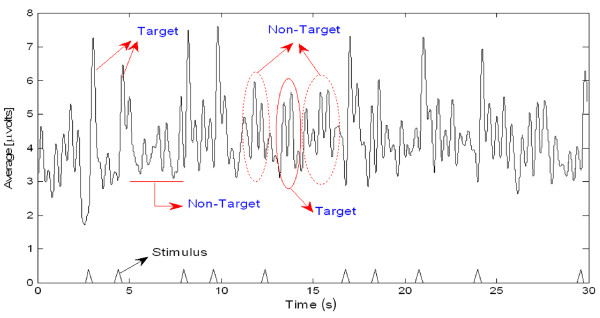
**The averages of eight-electrode data in Figure 8 (350 ms ISI): P300s are not detected in some periods**. By averaging, the amplitude of a target gets bigger compared to that of a non-target, if the signal is long time stationary. But, this will fail for dissimilar trials as indicated with the ellipse mark (i.e., solid circle for the target and dash circle for the non-target).

**Figure 13:**
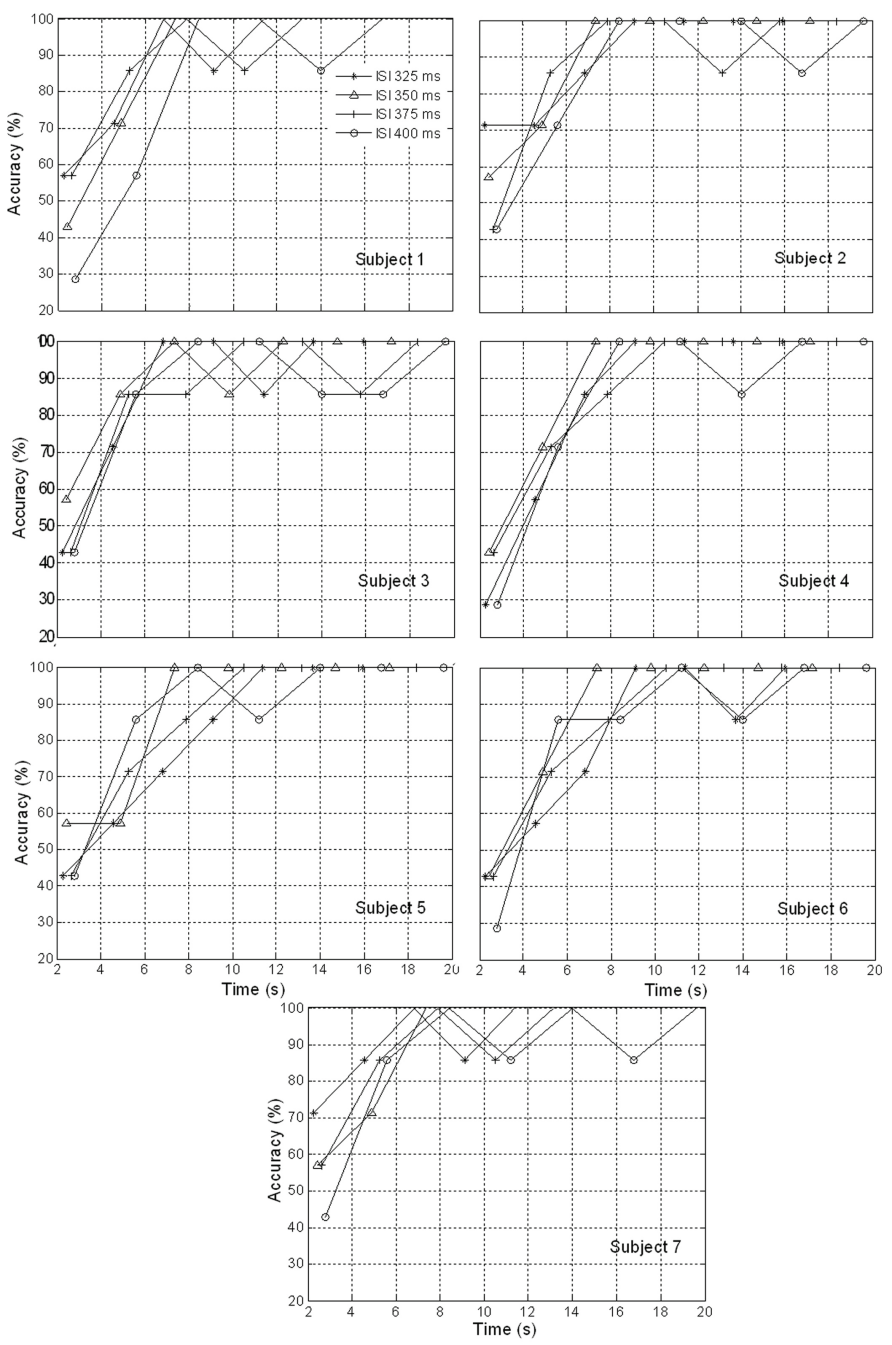
**Comparison of classification accuracies along seven stimuli (four ISIs, seven subjects): ISI 350 ms was the best out of four**. All subjects achieved an average classification accuracy of 100% after three blocks of stimulus presentations were averaged (i.e., 8 s). In this regard, the subject intention will be recognized after eight seconds of the first given stimulus.

Preparatory to an analysis of the features of P300 components from EEG signals in real-time, actual signals were recorded in an eight-channel (Fz, Cz, Pz, Oz, P7, P3, P4, and P8) configuration. Figure 3 shows the observed EEG signals with background signal amplitudes of around 300 micro volts. Figure 4 shows the pre-processed signals with amplitudes of around 25 micro volts, which were filtered using a sixth-order BPF with cut-off frequencies of 1 Hz and 12 Hz. One way of gaining further insights into EEG signals is by introducing ANPCA techniques. The present model of EEG analysis consists of four main steps: pre-separation (learning rate *β *of 0.6), whitening (forgetting factor *η *of 0.01), separation (forgetting factor *γ *of 0.002), and estimation (learning rate *α *of 0.3). In this algorithm, the pre-separation and the whitening steps enable faster adaptation at the separation step. The performance of the component separation of the ANPCA algorithm in the output was evaluated using (36). The evolutions of *PI*(*Ni*) for six different run of the proposed method generated from the data with 350 ms ISI is given in Figure 5. It can be seen that the algorithm takes between four and ten epochs to converge. Depending on the simulation run, the performance factor varies from -22 dB to -33 dB, due to random differences in the source signals. The robustness of the ANPCA was evaluated by comparing its separation performance with suggested algorithms (i.e., NPCA [45], Nonstationary Source Separation-Joint Diagonalization (NSS-JD) [42], Joint Approximate Diagonalization of Eigen-matrices (JADE) [46], and Second-Order Blind Identification (SOBI) [47]) as shown in Figure 6. Figures 7, 8, 9, and 10 show the real-time-extracted signals from eight-electrode of the P300 component using the ANPCA algorithm using ISI of 325 ms, 350 ms, 375 ms, and 400 ms, respectively. The P300 amplitudes of individual subject, taken from Fz electrode, for ISI of 325 ms, 350 ms, 375 ms, and 400 ms, respectively, is shown in Figure 11 (a) P300 amplitude upon a single stimulus and (b) P300 upon multiple stimuli. By averaging the eight extracted signals from the eight-electrode, the P300 components were not detected in some periods as indicated in Figure 12. This signal was averaged using the 350 ms ISI data. Comparative plots of the classification accuracies along seven stimuli for all subjects (subjects 1-7) are provided in Figure 13. The best classification accuracy was achieved using ISI 350 ms. The average value of the classification accuracies upon seven block stimuli for all of the subjects is given in Table 1. The classification using ISI 350 ms gave the the higher average value with smallest standard deviation.

**Table 1:** Average value of the classification accuracies upon seven stimuli

Subjects	ISI 325	ISI 350	ISI 375	ISI 400
S1	87.755	87.755	89.796	81.633
S2	89.796	89.796	87.755	85.714
S3	85.714	91.837	85.714	85.714
S4	81.633	87.755	85.714	83.673
S5	79.592	87.755	85.714	87.755
S6	79.592	87.755	85.714	83.673
S7	91.837	89.796	89.796	85.714
Average	85.131 ± 4.959	88.921 ± 1.607	87.172 ± 1.941	84.839 ± 1.992

## Discussion

The ability to measure and classify single-trial responses in real-time from specific brain regions has important theoretical and practical implications for both clinical and research applications. In this study, the amplitude of the background signal was around 300 micro volts as shown in Figure 3. Since the amplitude of the P300 component is very small (around 1.5 micro volts) compared with that of the background, the pre-processing filtering is required. These EEG signals were filtered using a sixth-order BPF with cut-off frequencies of 1 Hz (i.e., to remove the trend from low frequency bands) and 12 Hz (i.e., to remove unimportant information from high frequency bands), respectively. However, as shown in Figure 4, the signals nonetheless were corrupted by noises with background signal amplitudes of around 25 micro volts. Although there were some noticeable improvements, classification of the signals with respect to the given stimulus remained difficult. Therefore, an ANPCA-algorithm-based multilayer neural network model that can be used to analyzed complex P300 component from EEG signals in real-time is proposed. The MNN model with back-propagation training algorithm has five layers: the input and output layers have the same number of units *N*; the first and third layers are nonlinear (a sigmoid function as a universal approximation), and the second and fourth layers are linear. Layer 2 contains *M *units, that is, as many as there are nonlinear PCs. The activations of the neurons in Layer 2 are the nonlinear PCs of the input data. The back-propagation algorithm with an adaptive learning rate and momentum was used to train the neural networks. The values of the learning rate and the momentum were estimated by trial and error until no further improvement in the performance index could be obtained. The parameter values chosen were 0.3 and 0.8, respectively. The networks were trained before the EEG signals are recorded for one session. The time length for the training was range from 15.925 s to 19.6 s for each ISI.

Figure 5 shows the evolution of *PI*(*Ni*) for six different simulation runs in one implementation of the proposed method. The performance of the ANPCA algorithm in (30) was evaluated using (36) with *W*(0) = *I*. A single block of *N *= 7000 samples has been used to compute all coefficient updates for six run, where $\hat{x}\left(k+Ni\right)=\hat{x}\left(k\right)$ for all integer values *i *≥ 0 and *i *≤ *k *≤ *N*-1. As it can be seen, the algorithm took between four and ten epochs to converge. Depending on the simulation run, the performance factor varies from -22 dB to -33 dB, due to random differences in the source signals. The accuracy of the method generally improves for increasing values of block length *N*. It can be confirmed that the ANPCA algorithm successfully separates the mixture of source signals. To evaluate the robustness of the ANPCA against background noise, the separation performance indices of the ANPCA were compared with the suggested algorithms (i.e., NPCA, JADE, NSS-JD, and SOBI). The accuracy of the recovered independent components compared to the sources was measured according to the specified performance function in (36). Figure 6 shows the overall performance of all algorithms. For data iterations longer than 5000 iterations, the performance index was not much better, but was more and more time consuming. The quality of separation increases dramatically after 1500 length of iterations for the proposed method (ANPCA) and after 4000 length of iterations for other algorithms. It's clear that the proposed method present the shortest iteration time performance index about little over 0.03 (an acceptable level for separation). Upon this, it is asserted that the ANPCA algorithm successfully separates mixed source signals. The same accuracy level of separation was achieved after 4000 iterations by using other algorithms.

The ICs that were produced from the observed data using the ANPCA algorithm (for ISI of about 325 ms, 350 ms, 375 ms, and 400 ms) are shown in Figures 7, 8, 9, and 10. Although the signals were still corrupted by noises (manifested as the high amplitudes of non-targets in some sessions), the behaviours of the extracted signals clearly represented the P300 components. The observed signal was of the P300 event-related potential signal form. For the ISI of about 325 ms (Figure 7), it was found that the amplitude of the P300 component was higher than for the other ISI, as shown in Figure 11 (a), but noisier than for the higher ISI. As noted in Figure 7, the non-target amplitudes were roughly similar to the target amplitudes. For the ISI of about 350 ms (Figure 8), the target and non-target amplitudes were clearer and easier to distinguish than for the other ISI. For the ISI of about 375 ms (Figure 9), it was found that in some sessions the non-target amplitudes were higher than the target ones. For the ISI of about 400 ms, it was found that none of the channels showed similar behavior, as indicated in Figure 10. In this case the assumption of long stationary segment for averaging method will cause loss of the time resolution. Figures 7, 8, 9, and 10 show that the extracted signal amplitudes decreased (i.e., from the Fz to the P8 channel) as the distance of the electrodes increased. Figure 11(a) plots the amplitudes of the P300 component for four different ISIs (Fz channel) upon a single stimulus (scale of 700 ms) and indicate that the short ISI could increase both a target and a non-target amplitudes. Figure 11(b) plots the amplitudes of the P300 component for four different ISIs upon multiple stimuli (scale of 60 s) and indicate the peak shifting of the P300 component with respect to the various ISIs. The experiment using 350 ms ISI showed the best performance. Figure 12 displays the averages of the signals extracted from the eight-channel with ISI 350 ms. By averaging, the amplitude of a target gets bigger compared to that of a non-target, if the signal is long time stationary. But, this will fail for dissimilar trials, as indicated in Figure 12 (i.e., solid circle for the target and dashed circle for the non-target). This is one of the main reasons why the proposed method does not use the averaging scheme.

Comparative plots of the classification accuracies for the seven subjects were provided in Figure 13. All subjects achieved an average classification accuracy of 100% after three blocks of stimulus presentations were averaged (i.e., 8 s). In this regard, the subject intention was be recognized after eight seconds of the first given stimulus. Shown alongside the average value of the classification accuracies upon seven block stimuli for all of the subjects, in Table I, are the corresponding 85% confidence intervals. According to Table 1, the experiment with ISI 350 ms provides the highest average classification accuracies (88.921%) and smallest standard deviation (1.807) over all subjects. By contrast, ISI 400 ms showed the worst classification accuracies (84.839%). However, the worst standard deviation (4.959) was given by the experiment with ISI 325 ms. These results reflect the fact that the best performance was obtained through the experiment with ISI 350 ms.

Routine P300 component of EEG signals has been widely used in the clinical circumstances [21-26]. In this context, the use of physiological signals rather than behavioral responses of patient are often advisable, albeit challenging. Overall, the P300 component has sparked considerable interest as a clinical-application diagnostic tool. The most efficient method of implementing the diagnostic tool is through real-time detection. The amplitude of different waveforms at a single point can also be displayed in a similar format. This type of display provides a more objective analysis of the EEG activity compared to a subjective visual analysis by a physician. Simultaneous video monitoring of the patient during the EEG recording is becoming more popular. It allows the physician to closely correlate EEG waveforms with the patient's activity and may help produce a more accurate diagnosis.

## Conclusions

The applicability of the proposed ANPCA method for extracting the P300 waves included in the EEG signals for real-time without down-sampling and averaging of the original signals was demonstrated. The separation performance factor of the ANPCA varied from -22 dB to -33 dB due to the randomness of source signals. In comparison with other algorithms (i.e., NPCA, NSS-JD, JADE, and SOBI), the ANPCA presented the shortest iteration time with performance index about 0.03. Since all the computations are done in real time, the ANPCA can be used as a viable tool for clinical applications.

## Competing interests

The authors declare that they have no competing interests.

## Authors' contributions

AT carried out data acquisition and processing and drafted the manuscript. KSH supervised the project and corrected the manuscript. MYJ provided suggestions to improve the manuscript. All of the authors read and approved the final manuscript.
